# Supplementary material for: Peptide Centric Vβ Specific Germline Contacts Shape a Specialist T Cell Response
Source: Front Immunol. 2022 Jul 29;13:847092. doi: 10.3389/fimmu.2022.847092 (PMC9372435; doi:10.3389/fimmu.2022.847092)
Supplement: Supplementary Figure 5 — Docking angles of Vβ2 and Vβ8 containing TCRs to pMHC. TCR3d_DA = Conventional docking angle; TCR3d_IA = Conventional incident angle (obtained from the TCR3d database: https://tcr3d.ibbr.umd.edu/); TRA = Angle formed from the TRA plane modeled by the TRA VJ center of mass and regression of CDR1-3a; TRA_germ = Angle formed from the TRA plane modeled by the TRA VJ center of mass and regression of only CDR1a and CDR2a; TRB = Angle formed from the TRB plane modeled by the TRB VDJ center of mass and regression of CDR1-3b; TRB_germ = Angle formed from the TRB plane modeled by the TRB VDJ center of mass and regression of only CDR1b and CDR2b; TCR = Angle formed from the TCR plane modeled by the TCR V(D)J center of mass and regression of CDR1-3a and CDR1-3b; TCR_germ = Angle formed from the TCR plane modeled by the TCR V(D)J center of mass and regression of only CDR1a, CDR2a, CDR1b, and CDR2b. Vβ2 (PDB IDs: 1FO0, 1KJ2, 1NAM, 2OL3, 6DFS, 6X31) are represented in orange and Vβ8 (PDB IDs: 3C5Z, 3C6L, 3RDT, 3RGV, 4N5E, 6DFW) TCRs are represented in cyan. Interaction angles between with pMHC between the Vb2 and Vb8 TCRs were compared, and significance was determined using Mann-Whitney U tests with Bonferroni multiple testing corrections. * denotes an adjusted p-value < 0.05 and ns denotes not significant. [file DataSheet_1.docx]

**Supplementary Information**

A B

C

**Supplementary Figure 1. Expansion, Vβ usage and clonal analysis of specific T cell populations after infection with *T. gondii* parasites.**

(A) Mice were infected orally with cysts from the OVA expressing Pru strain *T. gondii*, and the numbers of the indicated pMHC tetramer^+^ CD8 T cells were enumerated from the spleen using flow cytometry. (B) Vβ usage of gated tetramer^+^ splenic CD8 T cells was determined by flow cytometry. Vβ usage in total splenic CD8 T cells from the same mice is shown for comparison. Mice were infected i.p. with Pru strain *T. gondii* parasites and analyzed at 21 days post infection. Statistical significance was determined by a t-test (****p<0.0001). (C) L^d^-HF10 tetramer^+^ CD8 T cells were single-cell sorted from mice 3 weeks after i.p. infection with Pru strain *T. gondii* parasites and TCRα and TCRβ genes from individual T cells were sequenced as described^36^. Restricted TCRβ CDR3 length in L^d^-HF10 specific T cells from 2 individual mice. The Vβ2^+^ L^d^-HF10 specific T cells include 57 total sequences, from 22 distinct clones, of which 21/22 have a TCRβ CDR3 length of 13aa. The distribution of TCRβ CDR3 length in bulk CD8 T cells is provided for comparison.

Top view

Group B

Group A

| **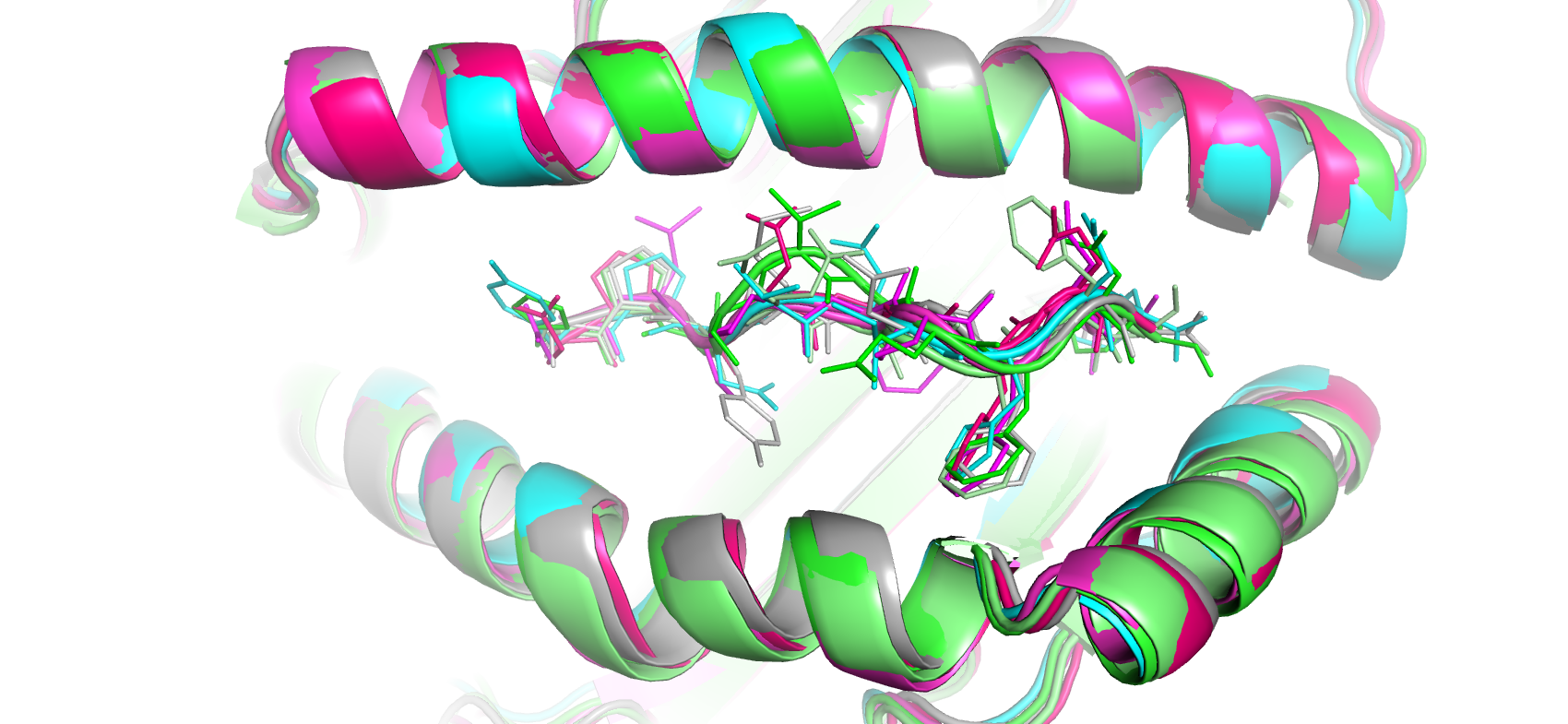 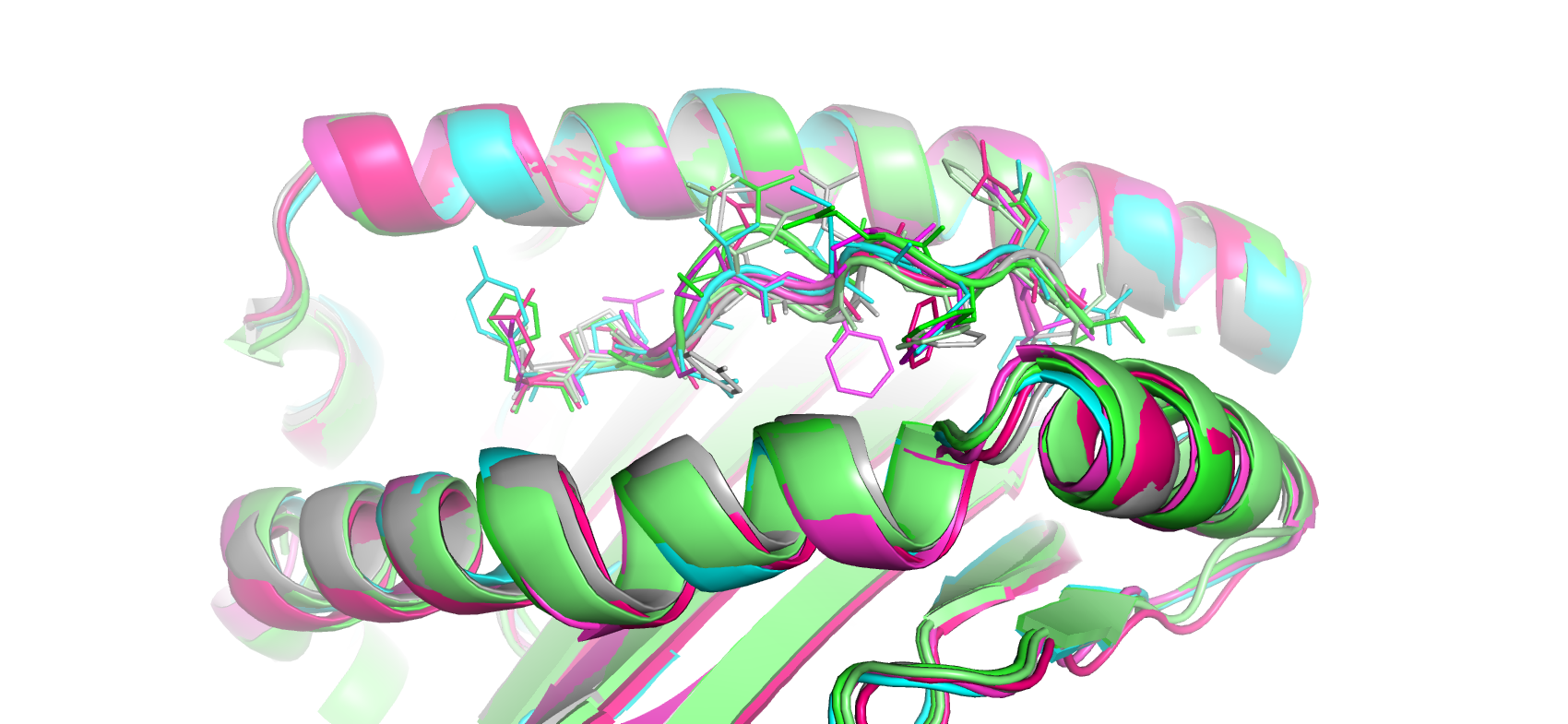**  α2 L^d^  α1 L^d^ | 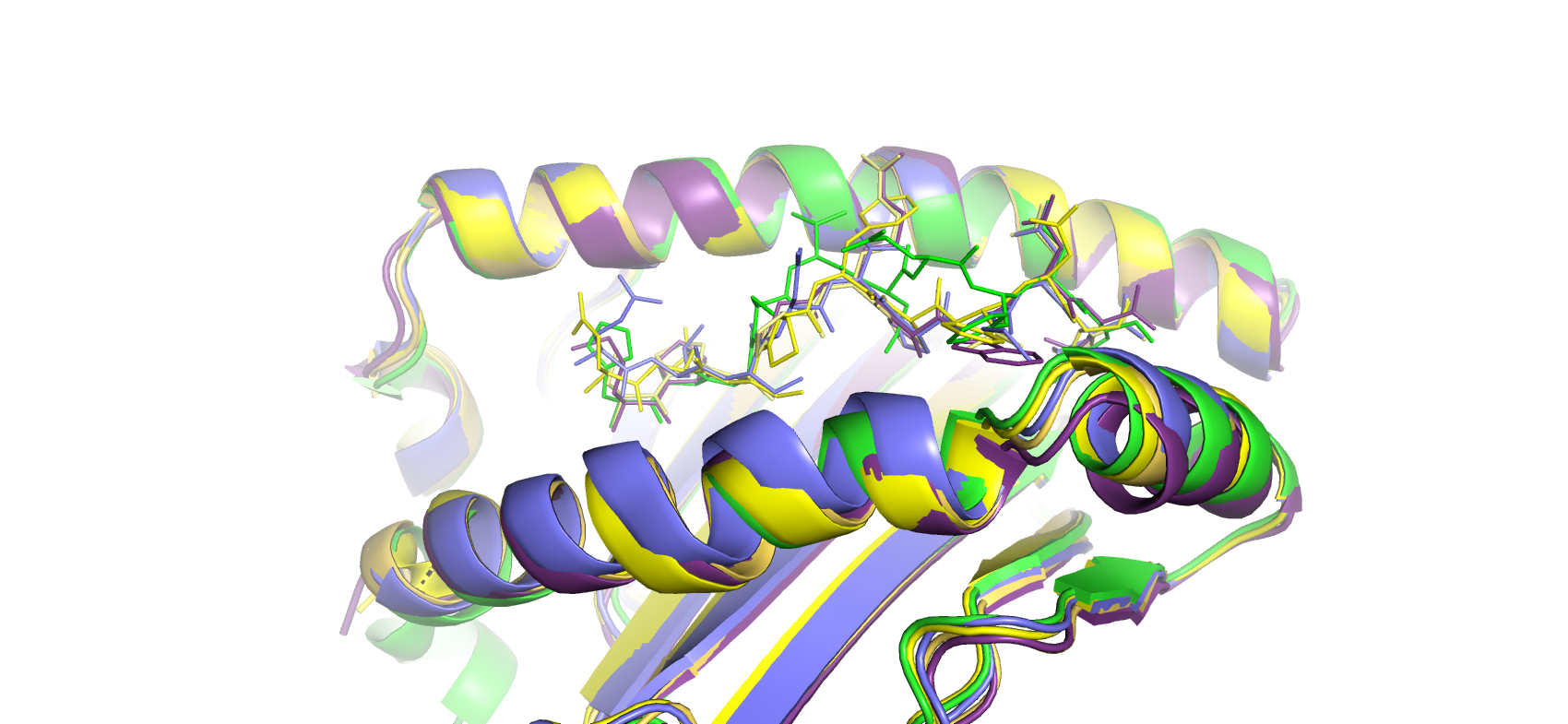  α2 L^d^  α1 L^d^  Side view  **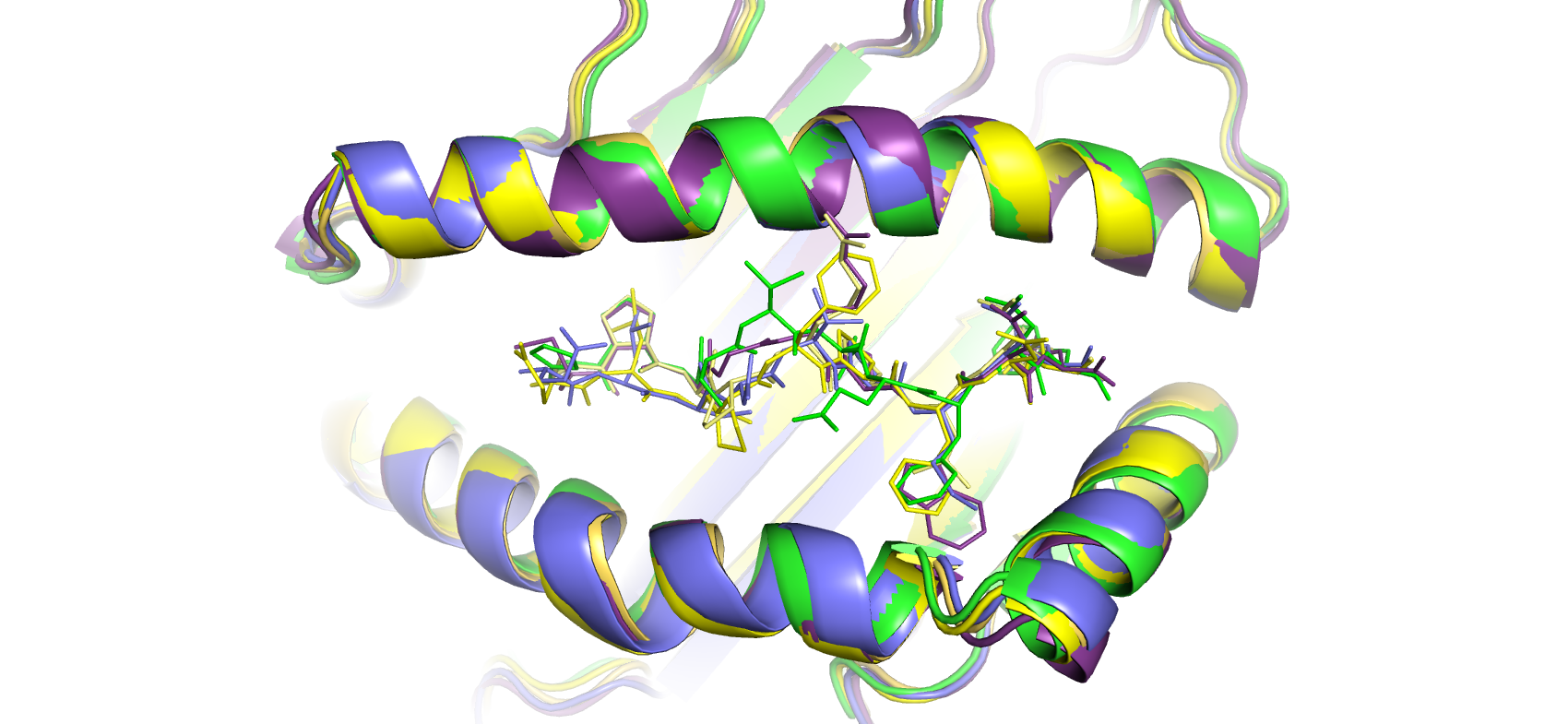** |
| --- | --- |

**
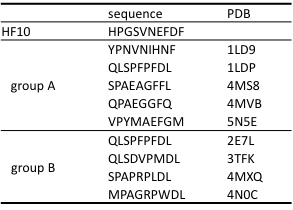
**

**Supplementary Figure 2. Conformation of peptides bound to H2-L^d^.**

Conformation of the bound HF10 peptide (green) in comparison to other L^d^-bound peptides (various colors). Peptides with a bend at p5 (HF10 and 5 other 9mer peptides in various colors) are on the left (group A), and peptides with a bend at p6/7 (HF10 and 4 other 9mer peptides in various colors) are on the right (group B). Upper panels show a top down view, and lower panels show a side view.

A C


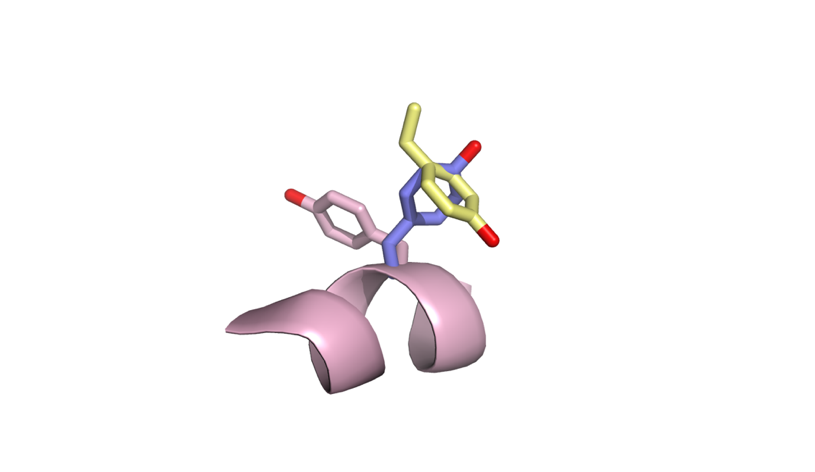


**TG6α** **99Y**

**L^d^ 155Y**

**103°**


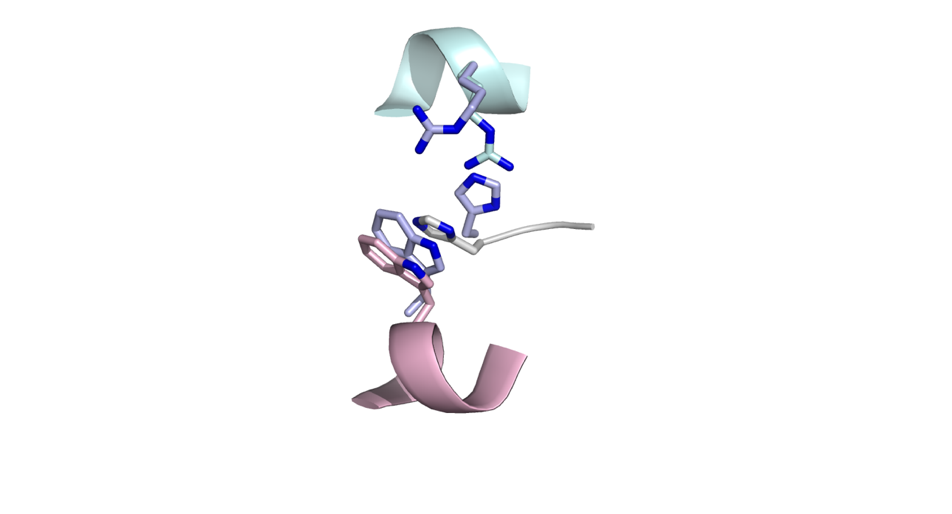


**L^d^ 62R**

**L^d^ 167W**

**HF10 p1H**

B  **CD25**  **CD44** D

**17°**

**92°**

**60°**

**Supplementary Figure 3. Binding and activation of TG6 TCR.**

**(A)** Corrected surface plasmon resonance (SPR) data for the binding of various concentrations of soluble L^d^-HF10 to ~2000 RU of biotinylated TG6 TCR immobilized in flow cell on SA sensor chip. Another flow cell contained the irrelevant biotinylated mouse BDC2.5 TCR to correct for the fluid phase SPR signal. Standard BIA evaluation 4 software was used to calculate the kinetic and equilibrium constants. **(B)** Naïve CD8 T cells from TG6 TCR transgenic mice were cultured with splenocytes incubated with WT HF10 peptide or the indicated alanine substituted variant peptides for 18 hours. Upregulation of surface CD25 and CD44 in response to TCR stimulation was measured by flow cytometry. **(C)** Ribbon representation of a portion of L^d^ α2 helix (light magenta) from the TG6/L^d^-HF10 complex shown with side chains of 155Y. Superimposed is the side chain of 155Y prior to TG6 TCR engagement (blue) and the side chain of 99Y (yellow) from the TG6 Vα CDR3. **(D)** L^d^ α1 (light magenta) and α2 helix (pale cyan) with side chains of 62R and 167W (light cyan and light magenta), and cartoon representation of a portion of HF10 peptide with side chain of pP1H (silver). Superimposed are the side chains of L^d^ 62R, 167W and p1H prior to TG6 TCR engagement (grey).

**
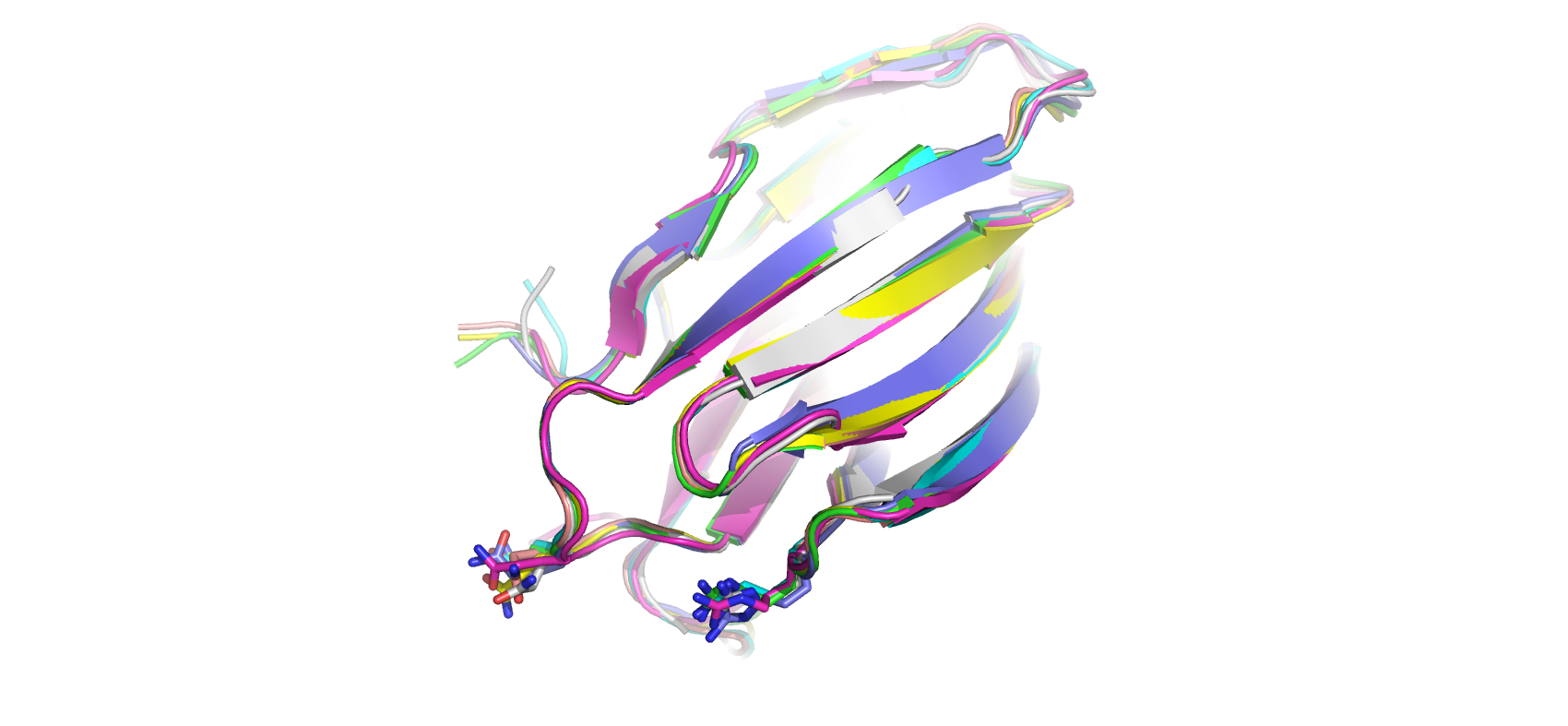
**A B


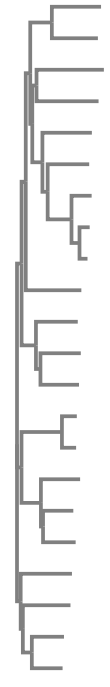


TRBV1: Vβ2

TRBV13.1

TRBV13.2 Vβ8

TRBV13.3

TRBV20

TRBV30

TRBV31

TRBV19

TRBV29

TRBV17

TRBV4

TRBV2

TRBV5

TRBV12.1

TRBV12.2

TRBV24

TRBV23

TRBV26

TRBV3

TRBV14

TRBV15

TRBV16

**50R**

**28Q**

PDB: TG6

PDB: 1FO0

PDB: 1KJ2

PDB: 1NAM

PDB: 2OL3

PDB: 1KB5

PDB: 6DFS

**
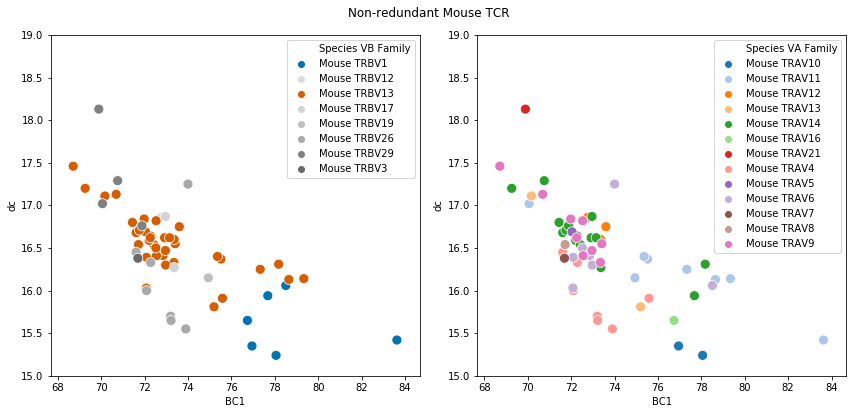
**C

Color coded by Vβ

Color coded by Vα

BC1 angle

**Supplementary Figure 4. Unusual CDR 1 and 2 loop conformation and Vα -Vβ interface geometry for Vβ2 containing TCRs.**

**(A)** Ribbon diagrams of published Vβ2 containing TCR structures. The positions of conserved germline contact residues 28Q and 50R are shown. **(B)** Tree showing relatedness of mouse TRBV genes based on inferred amino acid sequences. **(C)** Plots of TRangle parameters dc distance versus BC1 angle for TG6 TCR (indicated by arrow) compared to non-redundant TCR structures in the PDB. Right panel shows data color coded to indicate TCR Vα usage. Left panel shows the same data color coded to indicate TCR Vβ usage and is reproduced from Fig. 4 for comparison.


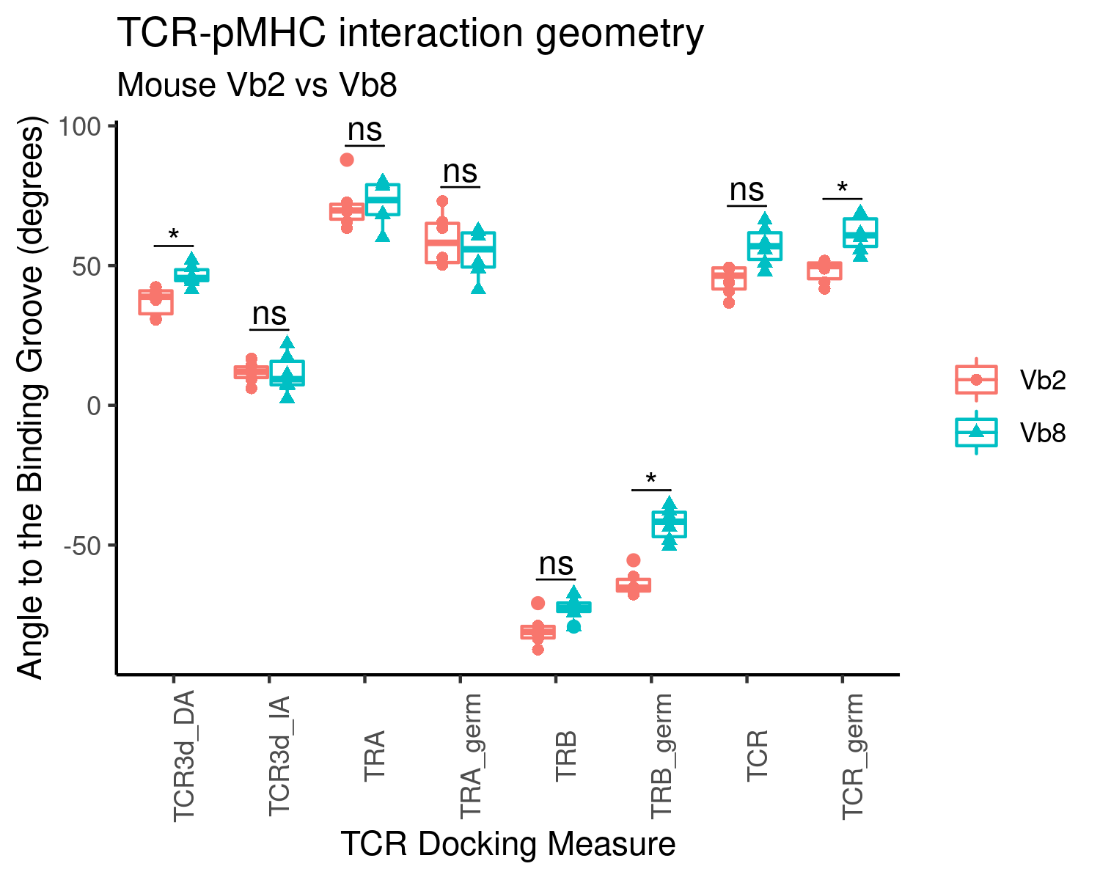


**Supplementary Figure 5. Docking angles of Vβ2 and Vβ8 containing TCRs to pMHC.**

TCR3d_DA = Conventional docking angle; TCR3d_IA = Conventional incident angle; TRA = Angle formed from the TRA plane modelled by the TRA VJ center of mass and regression of CDR1-3a; TRA_germ = Angle formed from the TRA plane modelled by the TRA VJ center of mass and regression of only CDR1a and CDR2a; TRB = Angle formed from the TRB plane modelled by the TRB VDJ center of mass and regression of CDR1-3b; TRB_germ = Angle formed from the TRB plane modelled by the TRB VDJ center of mass and regression of only CDR1b and CDR2b; TCR = Angle formed from the TCR plane modelled by the TCR V(D)J center of mass and regression of CDR1-3a and CDR1-3b; TCR_germ = Angle formed from the TCR plane modelled by the TCR V(D)J center of mass and regression of only CDR1a, CDR2a, CDR1b, and CDR2b. Vβ2 (PDB IDs: 1FO0, 1KJ2, 1NAM, 2OL3, 6DFS, 6X31) are represented in orange and Vβ8 (PDB IDs: 3C5Z, 3C6L, 3RDT, 3RGV, 4N5E, 6DFW) TCRs are represented in cyan. Interaction angles between with pMHC between the Vb2 and Vb8 TCRs were compared, and significance was determined using Mann-Whitney U tests with Bonferroni multiple testing corrections. * denotes an adjusted p-value < 0.05 and ns denotes not significant.

**TG6 (mVα4.9)**

**YAe62 (mVα4.12)**

**YAe62 (mVα4.12)**

**
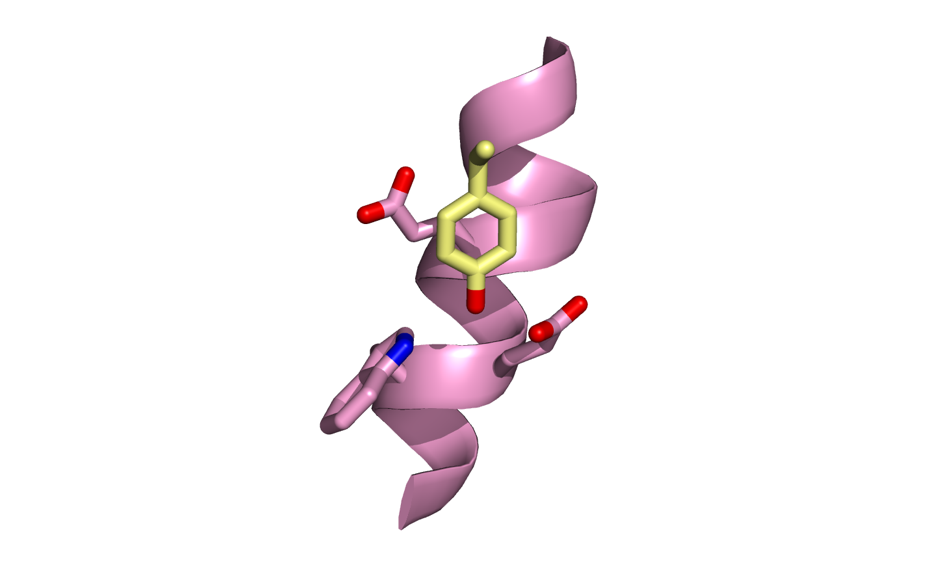

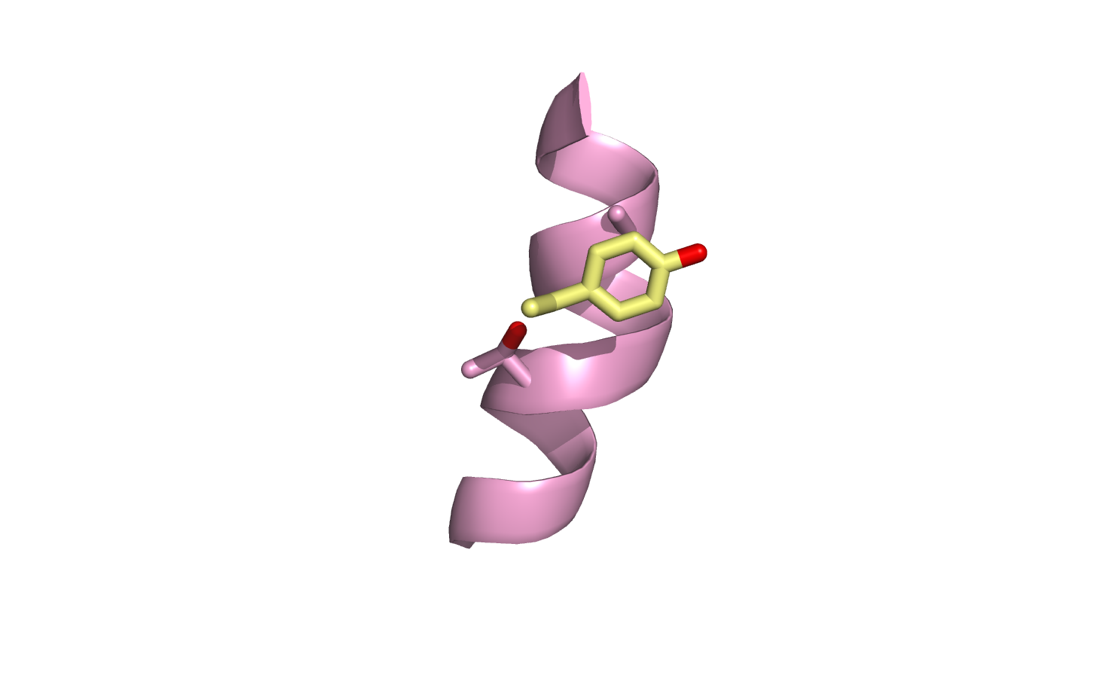
**
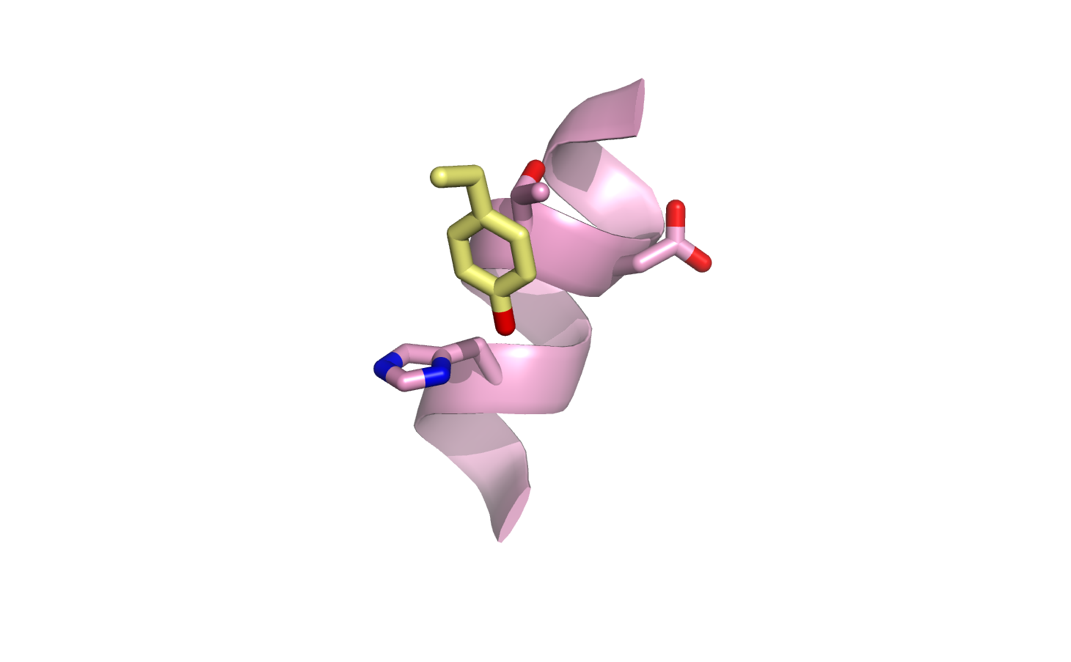


α158A

Vα29Y

α2 K^b^

α163T

Vα29Y

β1 IA^b^

β77T

β76D

β81H

Vα29Y

α163E

α166E

α167Y

α2 L^d^

**Supplementary Figure 6. Conserved germline contact between Vα CDR1 of TG6**

MHC is shown in purple ribbon with selected side chains shown, and the TCRα germline contact residue 29Y shown in yellow. Atoms are shown in CPK coloring. Two published TCR structures are shown for comparison.

**
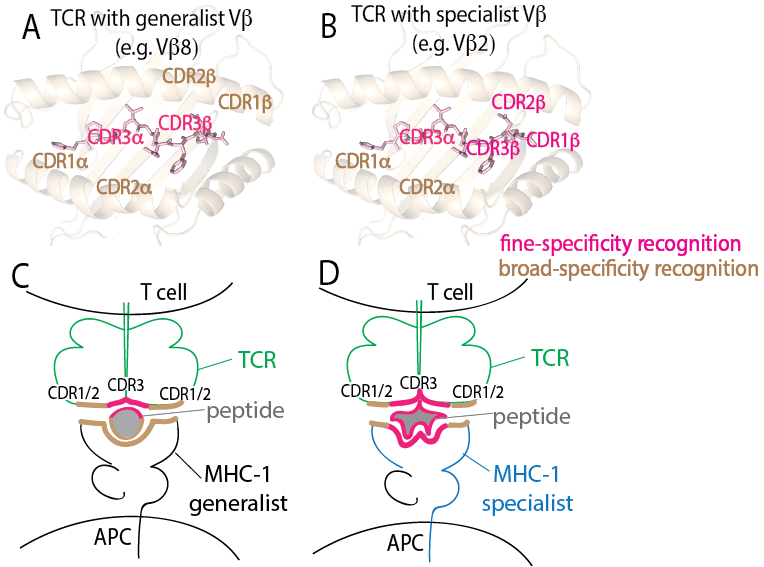
**

**Supplementary Figure 7. Generalist and Specialist Strategies in T cell Antigen Recognition.** (A-B) Interactions of TCR CDR loops with pMHC. MHC is shown as transparent brown ribbon diagram, and antigenic peptide is shown in pink stick, with positions of TCR CDR loops indicated. (A) For TCRs that contain generalist Vβ segments such as Vβ8, the highly variable CDR3 loops (purple text) are positioned over the peptide and provide for fine-specificity of ligand recognition, whereas the germline encoded CDR1 and 2 loops, (brown text), are positioned over the MHC, and provide for relatively broad recognition of different allelic forms of MHC. (B) For TCRs that contain specialist Vβ segments such as Vβ2, the TCRβ CDR loops are shifted away from the α1 helix of MHC, such that all 3 CDRβ loops can contribute to the fine-specificity for peptide. (C-D), Diagram of TCR-peptide-MHC interactions with regions of broad specificity indicated by thick tan lines, and regions of fine specificity indicated by thick purple lines. (C) Generalist MHC-I molecules bind broadly to peptides, such that most of the fine specificity for peptides comes from the TCR. (D) Specialist MHC molecules such as MHC-I L^d^ binding in a highly specific manner with peptide, such that the fine specificity for peptide is shared between the TCR and MHC.


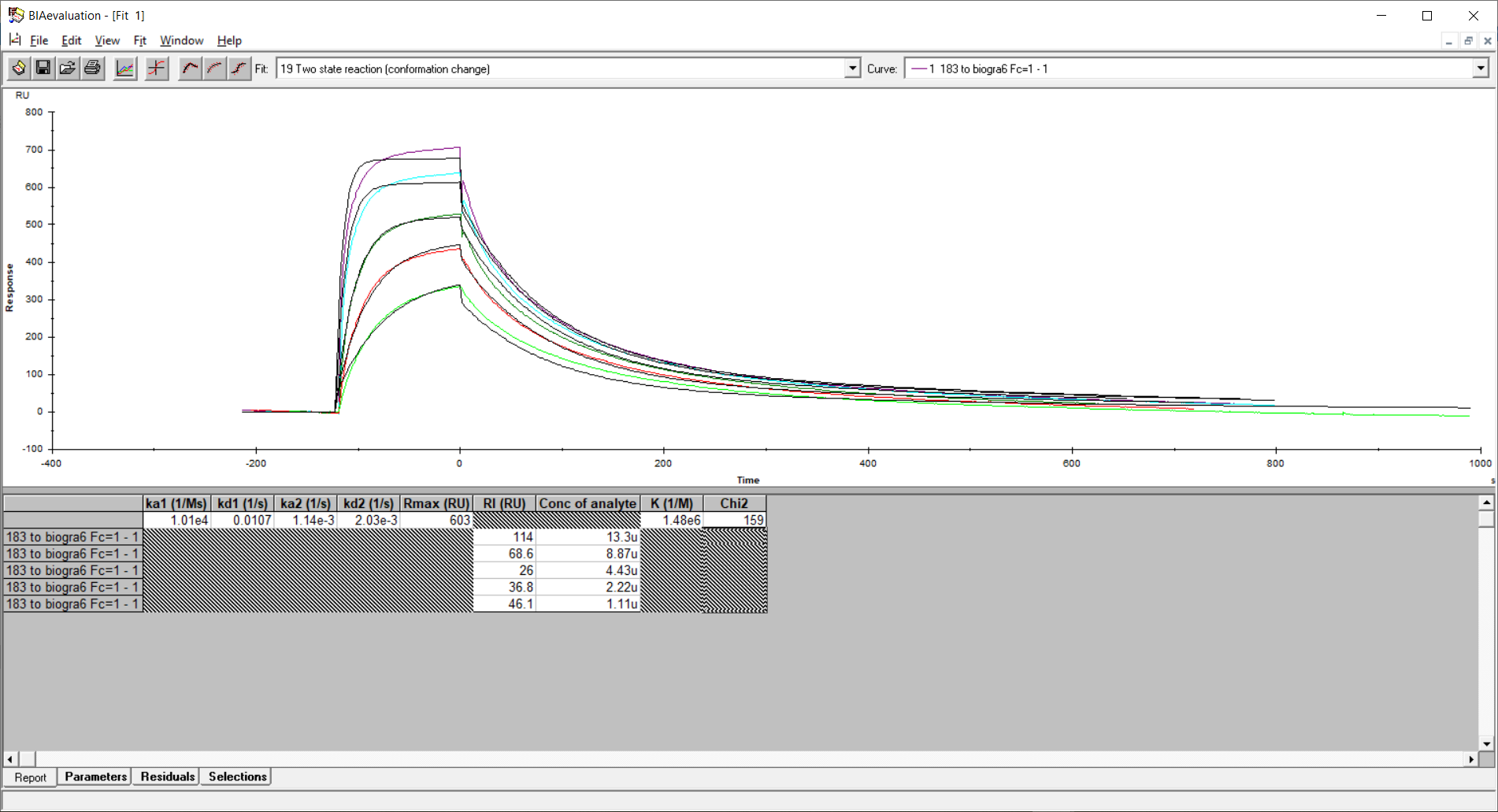


**Supplementary Figure 8. Biacore Binding of TG6 to WT Ld-HF10 data processing with BIAevaluation 4.1.1**

A B
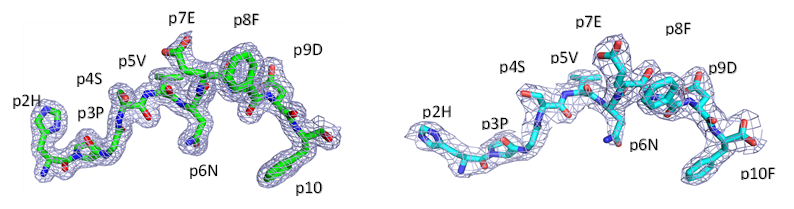


α2 L^d^

α1 L^d^

α1 L^d^

HF10

HF10

α1 L^d^

α2 L^d^


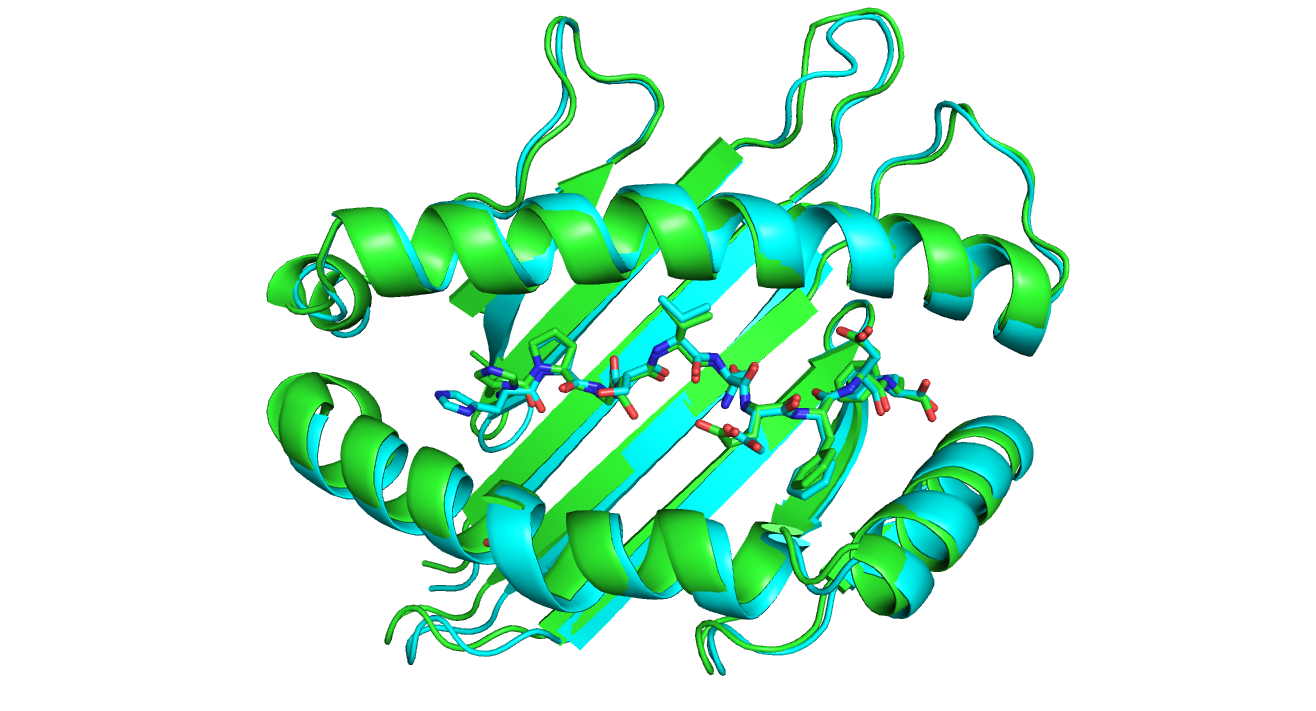


C

HF10

α2 L^d^

**Supplementary Figure 9. Structure comparison of covalent and Non-covalent Ld-HF10**

(A) Structure of covalent Ld-HF10 with the HF10 electron density shown. (B) Structure of non-covalent Ld-HF10 from TG6/Ld-HF10 complex with the HF10 electron density shown. (C) superposition of covalent Ld-HF10 (green carbon, CPK coloring) and non-covalent L^d^-HF10 structure (Cyan carbon, CPK coloring).

|  | **TCRα** | **TCRβ** |
| --- | --- | --- |
| **TG6 sequence** | TRAV6-7/DV9, TRAJ52*01, CALGDPTGANTGKLTF | TRBV1, TRBJ2-1*01, CTCSAGRGGYAEQFF |
| Mouse 1 (1) | TRAV6-7/DV9, TRAJ30*01, CALRIQDTNAYKVIF | *TRBV1, TRBJ2-1*01, CTCSAGRGGYAEQFF |
| Mouse 1 (2) | TRAV6-7/DV9, TRAJ53*01, CALSEGNSGGSNYKLTF | *TRBV1, TRBJ2-1*01, CTCSAGRGGYAEQFF |
| Mouse 1 (3) | TRAV6-5, TRAJ32*01, CPGSSGNKLIF | *TRBV1, TRBJ2-1*01, CTCSAGRGGYAEQFF |
| Mouse 2 (1) | TRAV6-7/DV9, TRAJ52*01, CALSDRDSGGSNYKLTF | *TRBV1, TRBJ2-1*01, CTCSAGRGGYAEQFF |
| Mouse 2 (2) | *TRAV6-7/DV9, TRAJ52*01, CALGDPTGANTGKLTF | TRBV1, TRBJ2-1*01, CTCSAGRGAHAEQFF |

**Supplementary Table 1. Clonal Analyses of TCR α and β Chains of GRA6-Specific T cells, Related to Figure 1**

L^d^-HF10 tetramer^+^ CD8 T cells were single-cell sorted from mice 3 weeks after i.p. infection with Pru strain *T. gondii* parasites and TCRα and TCRβ genes from individual T cells were sequenced as described^36^. The TG6 TCRβ coding sequence from the original L^d^-HF10 specific T cell hybridoma^8^ was found independently in 2 additional mice, each time paired with a closely related TCRβ. Likewise, the TCR α gene of the TG6 TCR was found independently in one additional mouse, paired with a closely related TCRβ gene. (* indicates the identical TG6 sequence found in a new clone.)

**Supplementary Table 2. Data Collection and Refinement Statistics, Related to Figures 2-6**

|  | H2-L^d^-HF10 | TG6 | H2-L^d^-HF10/TG6 complex |
| --- | --- | --- | --- |
| PDB code | 8D5N | 8D5P | 8D5Q |
| **Data Collection** |  |  |  |
| Space group | C2 | P2_1_2_1_2 | P4_1_ |
| Cell dimensions |  |  |  |
| *a*, *b*, *c* (Å) | 121.503 | 86.16 | 91.168 |
|  | 139.816 | 196.331 | 91.168 |
|  | 87.3441 | 63.112 | 106.247 |
|  |  |  |  |
| α, β, γ (°) | 90 | 90 | 90 |
|  | 130.824 | 90 | 90 |
|  | 90 | 90 | 90 |
| Resolution Range (Å) | 77-1.8 | 98-2.75 | 90-2.50 |
| *R*_sym_ or *R*_merge_*† | 0.09(0.53) | 0.01(0.1) | 0.03(0.29) |
| *I*/σ*I** | 9.6(1.3) | 16(3) | 26.6(1.6) |
| Completeness (%)* | 99.6(90.1) | 98.5(90) | 99.8 (98.4) |
| Redundancy* | 1.9(1.8) | 1.2(1) | 6.4(5.5) |

*All data (outer shell).

† Rmerge = Σ(jI −〈I〉j)/Σ(I).

|  | H2-L^d^-HF10 | TG6 | H2-L^d^-HF10/TG6 complex |
| --- | --- | --- | --- |
| **Refinement** |  |  |  |
| Resolution Range (Å) | 50-1.8 | 50-2.75 | 50-2.50 |
| No. reflections | 102152 | 26636 | 27675 |
| Rwork/ Rfree‡ | 19.91/22.66 | 23.89/29.45 | 17.16/21.79 |
| No. atoms | 6745 | 7152 | 5350 |
| Protein | 6267 | 7138 | 5118 |
| Ligand/ion | 98 | N/A | 210 |
| Water | 380 | 14 | 22 |
| B-factors |  |  |  |
| Protein | 37.62 | 35.92 | 68.59 |
| Ligand/ion | 63.95 | N/A | 57.927 |
| Water | 40.25 | 15.60 | 80.55 |
| R.m.s deviations |  |  |  |
| Bond lengths (Å) | 0.013 | 0.019 | 0.019 |
| Bond angles (º) | 1.664 | 1.934 | 1.932 |

‡ Rfree = ΣTestjjFobsj − |Fcalc|/ΣTest |Fobsj, where “Test” is a set of ∼5% of the total reflections randomly chosen and set aside.

**Supplementary** **Table 3. TG6 Binding Affinities to WT and Mutants HF10 Peptide, Related to Figure 3**

| Peptide mutations | ka1  (1/M·s) | kd1 (1/s) | ka2  (1/s) | kd2  (1/s) | Kd  (µM) |
| --- | --- | --- | --- | --- | --- |
| HF10 WT | 1.1X10^4^ | 0.014 | 0.002 | 0.005 | 0.42 |
| p1A | 5690 | 0.048 | 0.015 | 0.006 | 1.02 |
| p2A | 7980 | 0.066 | 0.009 | 0.014 | 1.84 |
| p4A | 1070 | 0.160 | 0.020 | 0.026 | 2.4 |
| p5A | 7510 | 0.284 | 0.009 | 0.014 | 1.85 |
| p6A | N/A | N/A | N/A | N/A | N/A |
| p7A | N/A | N/A | N/A | N/A | N/A |
| p8A | N/A | N/A | N/A | N/A | N/A |
| p9A | 52.7 | 0.175 | 0.008 | 0.009 | 170 |
| p10A | N/A | N/A | N/A | N/A | N/A |

Single HF10 residue alanine substitutions were tested to measure their effects on TG6 TCR binding.

**Movie Legends**

**Movie S1. Rotation of the L^d^-HF10 structure,** **Related to Figure 2**

The L^d^ α1-α3 sequence is shown as a silver ribbon diagram with the side chains of Trp97, Phe116, and Tyr156 shown. The bound L^d^ peptide is shown as a green stick diagram with anchor residues Pro2 and Phe10 shown in yellow and the Glu7 shown in red. Note the bend in the peptide to accommodate the aromatic residues in the peptide binding site. Also note that the 10-mer peptide fits compactly within the peptide binding groove, with only the side chain of Glu7 pointing away from the MHC molecule.

**Movie S2. Rotation of the TG6 TCR-L^d^-HF10 structure,** **Related to Figure 3**

The L^d^ α1-α3 sequence is shown as a silver ribbon diagram, the HF10 peptide is shown as a green stick diagram with anchor residues Pro2 and Phe10 in yellow and the primary TCR contact residue Glu7 in red. The TG6 TCR α and β chains are shown as red and tan ribbons respectively with the side chain of Arg97 shown in blue. Note the contact of Arg97 with Asp9 of the peptide and the close wrapping of the CDR3 loop around Glu7 of the peptide.

**Movie S3. Comparison of the TG6 and Yae62 TCR structures,** **Related to Figure 4**

The TG6 TCR (faint blue ribbon) is shown superimposed on the Yae62 (faint orange ribbon) with TCRα chains (very faint ribbons) aligned. The TCRβ CDR1 and 2 loops of both structures are in bold colors. Side chains of conserved germline contact residues are shown (Gln28 and Arg50 of TG6 TCRβ and Tyr48 of Yae62). Prolines 30 and 52 in CDR1 and 2 loops respectively of TG6 TCRβ are shown in dark blue. Note the displacement of the CDR1 and 2 loops between the 2 structures due to the distinct TCRα-β interface and CDR loop conformations.
